# On the solubility of azodicarbonamide in water/DMSO mixtures: an experimental and computational study

**DOI:** 10.1098/rsos.231831

**Published:** 2024-06-05

**Authors:** Giovanni Macetti, Luca Sironi, Costanza Rovida, Ilaria Geremia, Raffaella Soave, Leonardo Lo Presti

**Affiliations:** ^1^ Department of Chemistry, Università degli Studi di Milano, Via Golgi 19, Milano, 20133 Italy; ^2^ TEAM mastery S.r.l., Via Giuseppe Ferrari 14, Como, 22100 Italy; ^3^ Institute of Chemical Science and Technologies ‘Giulio Natta’, National Research Council of Italy, Via Golgi 19, Milano, 20133 Italy; ^4^ Istituto Nazionale di Fisica Nucleare – Laboratori Nazionali di Frascati, Frascati, Roma, Italy

**Keywords:** solubility, azodicarbonamide, mixtures, molecular dynamics, UV-Vis spectroscopy

## Abstract

This work aims at studying why azodicarbonamide (ADCA), a formally apolar compound with good hydrogen bond (HB) acceptors, is soluble only in polar aprotic solvents like dimethyl sulfoxide (DMSO) but not in water. Solubility measurements, as well as quantum mechanical and classical molecular dynamics simulations, were employed to tackle the problem. We found that in the liquid phase a polar conformer of ADCA (*µ* = 8.7 D), unreported to date, is favoured under the enthalpic drive provided by a highly polar solvent. At the same time, the very high hydrogen bond propensity of water with itself prevents this solvent from providing an effective hydrogen bond-mediated solvation. Solvents bearing good HB acceptors, while lacking strong HB donors, contribute to further stabilizing solute–solvent adducts through weak and fluxional HBs that involve the amide groups of ADCA. Implications for the solubility of ADCA down to *µ*M concentrations were evaluated, also with the aid of classical simulations of solution nanodroplets.

## 1. Introduction

**Scheme 1.**
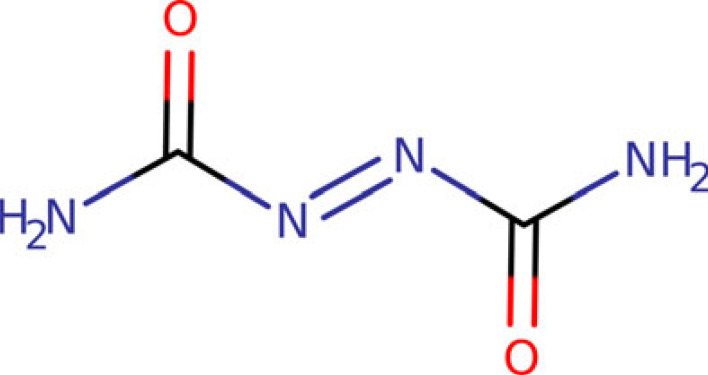
Azodicarbonamide structural formula.

Azodicarbonamide (ADCA, C_2_H_4_N_4_O_2_, MW = 116.080 g mol^−1^, CAS No. 123-77-3, Scheme 1) is the diamide of the azodicarboxylic acid, commercially available as a dark yellow crystalline powder. This compound used to be largely employed in industrial application as a bleaching agent and dough conditioner in food industry [1], but it is now banned in the EU and Australia for use in human foods due to suspect toxicity of its side products, such as semicarbazide (CH_5_N_3_O) [2,3]. Even though ADCA is still employed in the production of foamed plastics [4], it was classified as a respiratory sensitizing agent (H334: May cause allergy or asthma symptoms or breathing difficulties if inhaled) as for CLP (Classification, Labelling and Packaging) Regulation (EC) No. 1272/2008, generating concerns especially at chemical industries where it is manufactured and used [5]. Accordingly, in the EU it is now listed as a substance of very high concern (SVHC) according to the REACH (Registration, Evaluation, Authorization and Restriction of Chemicals) Regulation (EC) No. 1907/2006 [6].

ADCA solubility is very low in most common protic and aprotic organic solvents, for instance in ether [7] and ethanol [8]. In particular, ADCA is very poorly soluble in water [9], while the solubility in dimethyl sulfoxide (DMSO) amounts to several mg ml^–1^ at room temperature [10]. The reasons are unclear, though, due to the apolar nature of the title compound, which also bears two carbonyl groups that may act as good HB acceptors (Scheme 1) [11].

The crystal structure of ADCA was first reported by Bryden in 1959 (CCDC AZDCAR) [12]. A more recent and accurate X-ray structure determination is also available (CCDC AZDCAR01) [13]. The only known polymorph is monoclinic, space group P2_1_/c, with ½ molecule in the asymmetric unit and 2 formulae in cell. The molecules always adopt a trans conformation with respect to the azo double bond. The molecular point group, C_2h_ (2/m), is not compatible with a net molecular dipole moment and the structure is actually apolar. The amide hydrogens are not pyramidalized, implying a sp^2^ hybridization of the terminal nitrogen atoms and poor acid-base properties. Accordingly, an extended delocalized π system is set up across the molecular backbone, accounting for the light absorption properties in the visible window of the electromagnetic spectrum.

The aim of this study is to understand the physico-chemical properties responsible for the solubility behaviour of ADCA in water and DMSO. To this end, we combine experimental and computational techniques. Accurate solubility measurements in water:DMSO mixtures under thermodynamic control were carried out using UV-Vis spectroscopy. The results were complemented by quantum and classical molecular dynamics calculations, which provided molecular-level insights to explain the macroscopic behaviour of the title compound. The simulation of nanodroplet-dispersed ADCA solutions, also in the presence of DMSO in traces, provides further hints on solute-solvent interactions.

## 2. Methods

### 2.1. Solubility measurement

Reagent-grade DMSO was purchased by Merck and used without further purification. In-home produced mQ water (*ρ* ∼ 9 µS cm^–1^) was used throughout. Pure ADCA, in the form of very fine yellow powder, was provided by Hebron S.A., Barcelona (batches AZ VI-50 L-2G59E and AZ 50-50 I 2J59E).

The experimental methodology proposed by Sacchi *et al*. [14] was applied throughout. Complete details can be found in the supporting information. The solubility of the title compound was determined by measuring the concentration of azodicarbonamide (ADCA) in saturated solutions using UV-Vis spectroscopy, using a Beckman Coulter DU–800 spectrophotometer equipped with D_2_ and W sources, in conjunction with quartz high performance cuvettes (QS grade, transparent down to 200 nm) with optical length of 1 cm. The maximum error on the measured absorbance should be lower than 5·10^–5^.

Initially, a fixed volume of solvent (10 ml) was placed in deep contact with an excess of finely ground powdered ADCA by continuous stirring for 1 h at the desired temperature. Then, the suspension was kept at rest for 30 min at the same specified temperature, or directly hot-filtered if very low ADCA concentrations were expected, typically in water-rich mixtures (see electronic supplementary material, section S1 in SI). In any case, the (supernatant) liquid was sampled and its absorbance immediately probed in the soft UV region of the spectrum. If necessary, the sampled aliquot was diluted to ensure that the measured absorbance fell within the linear range of the detector (see Supplementary Information for further details). The contribution of the solvent was subtracted as a blank before any quantification.

The maximum UV absorbance of ADCA occurs at 242 nm [15], which falls below the UV cutoff limit of DMSO (265 nm) [16]. According to available literature protocols for the analysis of ADCA in DMSO/dimethylformamide mixtures [15,17], the wavelength of 272 nm was selected as the best one in terms of tradeoff between removing solvent interference and achieving a sensitive detection. Recent studies aiming to accurately quantify traces of ADCA in various matrices have also used similar wavelengths, such as 276 nm or 280 nm [17,18]. Measurements were carried out in five chemical environments, starting from pure DMSO (D100, where D refers to DMSO and the number indicates its percentage fraction in the mixture) to pure water (D000), with intermediate DMSO:water mixtures at 80:20 (D080), 50:50 (D050) and 20:80 (D020) compositions. The solubility of ADCA in each solvent mixture was investigated in a nominal temperature range of 19–39°C, with 5°C increments.

### 2.2. Single-molecule quantum calculations

First-principles all-electron simulations were conducted using the correlated Møller-Plesset expansion truncated at second order [19,20], as implemented in the Gaussian 09 code [21]. The triple zeta Pople's 6–311G(d,p) basis set was chosen to ensure an accurate representation of the valence shell electrons. The geometries of all the conformers were fully optimized in the gas phase in C_1_ symmetry. No imaginary frequencies were detected in correspondence to the stationary points. To account for the solvent, the Solvation Model based on Density (SMD) approach by Cramer & Truhlar was also employed [22]. The solvents considered, along with their respective dielectric constants, were DMSO (*ε* = 46.83) and water (*ε* = 78.35).

### 2.3. ADCA and ADCA/solvent molecular dynamics

#### 2.3.1. Liquid ADCA

An initial simulation box of liquid ADCA (686 molecules) was generated starting from the crystallographic structure [13] with the Boxliq routine of the MiCMoS package [23,24]. To dispose of hard contacts, 2 million steps of Monte Carlo were performed. This was followed by 400 ps of preliminary classical dynamics at *T* = 500 K, employing the Lennard–Jones–Coulomb (LJC) potential [25] to ensure equilibration. The final frame of the simulation was then used in GROMACS 2018.4 [26] to perform 1 ns-long dynamics under NpT conditions. The OPLS-AA force field [27,28] was employed throughout. Geometry descriptors were parametrized using quantum simulations at the B3LYP/6–31G* level of theory, employing the Automated Topology Builder (ATB) webserver [29], with a minimum editing of torsional parameters. A Berendsen barostat and a stochastic Bussi-Donadio-Parrinello thermostat [30] were employed to restrain the thermodynamic variables to their reference values (1 bar, 500 K). The leap-frog integrator was used with a short time step of 0.5 fs to avoid divergence at high temperature. All the covalent bonds were constrained to their equilibrium lengths through the LINCS algorithm [31], while the Particle Mesh Ewald (PME) method [32] was used to account for long-range electrostatics in conjunction with non-bonded interaction cutoff of 10 Å. Full periodic boundary conditions were exploited, starting from a 4.6 nm large cubic box.

#### 2.3.2. ADCA solutions

We simulated solutions of ADCA in DMSO and water with a concentration of approximately 0.5 M, exploiting the same procedure described in §2.3.1 above to run 1 ns-long NpT trajectories at *T* = 298 K with the OPLS-AA force field, as implemented in GROMACS 2018.4. Full information on the preparation of the starting simulation boxes is given in the supporting information (electronic supplementary material, section S4 SI), together with examples of input topologies (electronic supplementary material, section S6 SI). Whenever present, water was modelled in an SPC [33] fashion. For testing purposes, some simulations were repeated also with the TIP4P water [34], obtaining identical outcomes within the numerical accuracy. An ADCA-free approximately 28% *w*/*w* DMSO:water mixture (close to the D020 solvent system, see §2.1) was also simulated under the same conditions, for comparison purposes (see electronic supplementary material, section S4.1.6 SI).

#### 2.3.3. Nanodroplets of ADCA solutions

Two models were considered. The ‘Water-DMSO-ADCA’ (WDA) one consisted of a liquid core of 37 ADCA molecules embedded in a droplet of 741 DMSO molecules. The core solution was bathed by 8170 water molecules. Overall, the simulated solvent mixture had a DMSO concentration of approximately 28% in weight, that is, reasonably close to the composition of the D020 solvent system (see §2.1 above). Very low DMSO concentrations were also considered to interact with the ADCA nanodroplet, namely either 2% (DMSO Diluted WDA, acronym DD-WDA, with 38 DMSO) or 0.2% (Highly DMSO Diluted WDA, acronym HD-WDA, with 4 DMSO). The starting point was the same as the WDA model, after a random removal of as many DMSO molecules as required to match the desired concentration.

**Figure 1.**
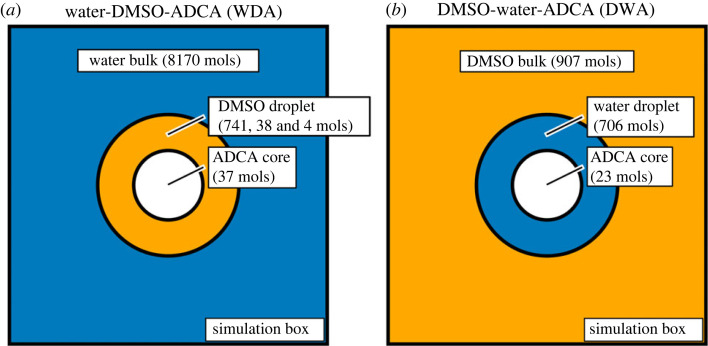
Water-DMSO-ADCA (WDA, left) and DMSO-Water-ADCA (DWA, right) three-component schemes. Both models are made by a core of ADCA molecules, surrounded by a droplet of solvent (WDA: DMSO or DWA: water) and immersed in the bulk solvent (WDA: Water or DWA: DMSO). Numbers in parentheses indicate the number of molecules in the selected model. The three different numbers for DMSO in the WDA model refer to the standard WDA, diluted WDA (DD-WDA) and highly diluted WDA (HD-WDA) models described in §2.

The second model labelled ‘DMSO-water-ADCA’ (DWA) accounts for the other compositional extreme. DWA consists of a roughly spherical nanodroplet of 23 ADCA molecules, surrounded by a droplet of 706 water molecules, in turn embedded in a bulk of 907 DMSO molecules. The simulated solvent mixture has a composition close to the D080 system (see §2.1 above), with a weight concentration of DMSO close to approximately 85%. At variance with WDA, where ADCA is kept initially in close contact with DMSO, DWA starts with ADCA embedded in a water shell. Both boxes were evolved at 298 K under NpT conditions for 10 ns at time steps of 0.5 fs with the OPLS-AA Force Field, as implemented in GROMACS 2018.4 [35]. Schematic representations of three-components ADCA nanodroplets solutions are shown in figure 1.

#### 2.3.4. Solvation free energy

For the two solvents considered (DMSO and water), calculations of free energies of solvation were carried out with the Bennet Acceptance Ratio (BAR) method [26], as implemented in GROMACS. A coupling parameter *λ* was varied stepwise from 0 (solute–solvent not interacting) to 1 (one ADCA molecule fully solvated) at steps of 0.05 for both van der Waals and electrostatic interactions. To generate the starting simulation box, we first equilibrated solutions at the nominal concentration of 0.2 mol l^–1^, using the same procedure detailed in electronic supplementary material, sections S4.1.1-S4.1.2 SI. Then, we erased all the solute molecules but one, and we re-equilibrated the liquid with the steepest descent minimization algorithm, followed by NVT and NpT preliminary dynamics runs, each one lasting for 100 ps. Eventually, a total of 21 runs of stochastic Langevin dynamics were carried out, each one with a duration of 1 ns and a time step of 2 fs. The non-bonded energy cutoffs were increased to 12 Å, while soft core potentials were active during the decoupling procedure. Like previous simulations, an isotropic Berendsen barostat and a Bussi–Donadio–Parrinello thermostat [30] were used to maintain a pressure of 1 bar and a temperature of 298 K.

## 3. Results

### 3.1. Solubility measurements

**Figure 2.**
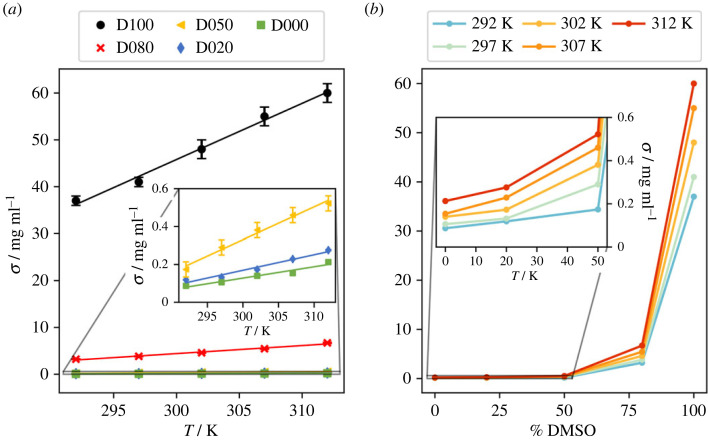
(*a*) ADCA solubility (*σ*, in mg ml^−1^) at equilibrium as a function of T and solvent mixture fractions. vertical bars correspond to 1 estimated standard deviation; if not visible, the bars have extension comparable with the dimension of the spot. The acronyms in the legend are explained in §2.1 and refer to the percentage of DMSO in the solution. Relevant regression parameters are shown in electronic supplementary material, table S3. (*b*) solubility data for ADCA in mixed DMSO:water solutions, plotted as a function of the solvent composition (% of DMSO) at constant temperature. The lines serve only as guides for the eye.

Contrasting data are present in the literature on quantitative estimates of solubility of ADCA [35,36]. Moreover, to the best of our knowledge, there is a lack of comprehensive literature information regarding protocols for accurate measurements of the solubility of pure ADCA. However, there is a general consensus on the fact that the solubility is quite high in DMSO (approx. 23 mg ml^–1^ at *T* = 25°C) [37,38] and very low in water (approx. 35 µg ml^–1^ at *T* = 20°C) [9]. The solubilities estimated in this work (figure 2, electronic supplementary material, table S2) are systematically higher than those available in public repositories but, as expected, they correctly span three orders of magnitude. On a qualitative level, we confirm that at 20°C the solubility is very low (< 0.1 mg ml^–1^) in water and good (> 30 mg ml^–1^) in DMSO. Our measurements are carried out under thermodynamic control conditions, i.e. ensuring complete equilibration of the solid/liquid mixtures. This implies that our estimates quantify the maximum amount of ADCA that can be dissolved in a specific solvent mixture at a given temperature.

As expected, the solubility of ADCA increases with temperature, indicating that the dissolution process is endothermic. The least-squares fitting of *σ*/T data shows that the solubility *σ* increments with a reasonably linear relationship in the 20–40°C range of T (figure 2*a*). The fitting quality slightly decreases (*R*^2^ < 0.98) in the low solubility environments such as D020 (20% DMSO) and D000 (pure water) due to the increased statistical fluctuations associated with very small *σ* values. Moreover, the slope of the linear functions exhibits a decreasing trend from 1.20(6) mg ml^–1^ K^–1^ in DMSO to 0.0060(8) mg ml^–1^ K^–1^ in water (electronic supplementary material, table S3). The higher the *σ* value, the steeper the increase in solubility with respect to the temperature (figure 2*a*).

The impact of solvent composition is substantial. The solubility increases monotonically with the amount of DMSO (figure 2*b*). This behaviour is more pronounced at elevated temperatures (indicated by hotter colours in figure 2*b*), although it is qualitatively consistent across all the temperatures. Starting from pure water, the addition of 20% DMSO results in approximately 30–40% increase in solubility, even though it remains in the range of 100–200 µg ml^–1^ (see also electronic supplementary material, table S2). On the other hand, the inclusion of 50% DMSO implies a *σ* increment of more than 100% with respect to pure water, up to approximately 0.2 mg ml^–1^ at 20°C and approximately 0.5 mg ml^–1^ at 40°C. Overall, up to a DMSO concentration of 50%, the solubility trend is roughly exponential. On the contrary, in solvent mixtures with a higher proportion of DMSO, *σ* grows even faster. For instance, the transition from 80% DMSO to 100% DMSO results in a tenfold increase in the solubility (electronic supplementary material, table S2).

In summary, DMSO is the optimal solvent for ADCA. Water, on the other hand, acts as an antisolvent. The addition of 20% of water from pure DMSO results in a tenfold reduction of *σ*, although the solubility remains on the order of several (3–7) mg ml^–1^. The solubility drops below 1 mg ml^–1^ in 1 : 1 water:DMSO mixtures and quickly tends to the limit of 0.1–0.2 mg·mL^–1^ when the amount of water further increases.

### 3.2. Conformational analysis

ADCA is insoluble in apolar solvents such as toluene or acetonitrile, while it is mildly soluble in highly polar aprotic solvents, such as DMSO (molecular dipole moment of about 4 D) [39] and hexamethylphosphoramide (molecular dipole moment of about 5 D) [40]. This observation suggests the involvement of a polar conformer of the azodicarbonamide in the solvation process.

**Figure 3.**
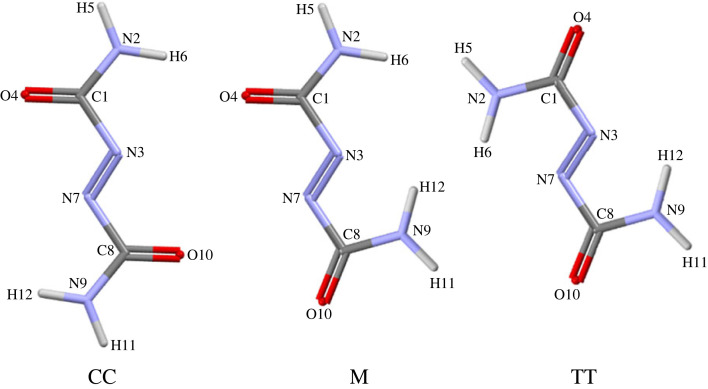
Symmetry-independent conformers of ADCA that correspond to stationary points of the quantum potential energy surface. ‘C’ means cis- with reference to terminal atoms of the sequence O–C–N = N, ‘T’ trans- and M stands for ‘mixed’ (one bond cis-, one trans-).

To investigate this hypothesis, we performed a conformational analysis of the isolated ADCA molecule at the MP2 level of theory (see Methods). ADCA possesses four rotatable bonds (Scheme 1, figure 3). These include the rotation of the terminal amide –NH_2_ groups around their N–C axes, as well as the rotation around the C1–N3 and C8–N7 bonds, which determine the relative rotation of the entire amide groups (figure 3). Isomerization around the central N = N double bond is expected to have a high kinetic barrier and was not considered in the conformational analysis. Consequently, the two amide groups were consistently kept in a trans configuration with respect to the double bond. The dihedral rotations around C1-N3 and C8-N7 were the only relevant factors to influence the molecular conformation (figure 3).

We selected the dihedral angle across the O–C–N = N atom sequence (figure 3) as a suitable conformational descriptor. The presence of extended π conjugation favours planar configurations, which indeed correspond to stationary points on the molecular potential energy surface. This implies that each O–C–N = N dihedral can adopt either a 0° (‘C’ conformation) or 180° (‘T’ conformation) value. Depending on what combination is observed, the molecule is said to assume a ‘CC’ (the conformation found in the crystalline phase), ‘TT’ or ‘M’ (mixed, ‘CT’ or ‘TC’) conformation (figure 3). In the gas phase, the TT conformer is more stable than CC by 8.4 kJ mol^–1^, while the M conformer does not correspond to an energy minimum (see electronic supplementary material, table S4). Unlike in the crystal phase, the CC and TT gas-phase conformers are not completely planar, as the dihedral angle across the O–C–N = N atom sequence amounts to 9.7° (CC) and 15.4° (TT).

The inclusion of solvent as a continuum dielectric (see Methods) allowed us to investigate the effect of the chemical environment. The calculations performed in water and in DMSO predicted the M conformer as the most stable (electronic supplementary material, table S4). It is worth noting that both the CC and TT conformers are poorly polar, with dipole moments not exceeding 1.8 D, while the two symmetry-equivalent M conformers possess a detectable dipole moment of approximately 8.7 D in both solvents. Interested readers can find full details in the supporting information.

### 3.3. Molecular Dynamics: liquid ADCA and two-component systems

**Figure 4.**
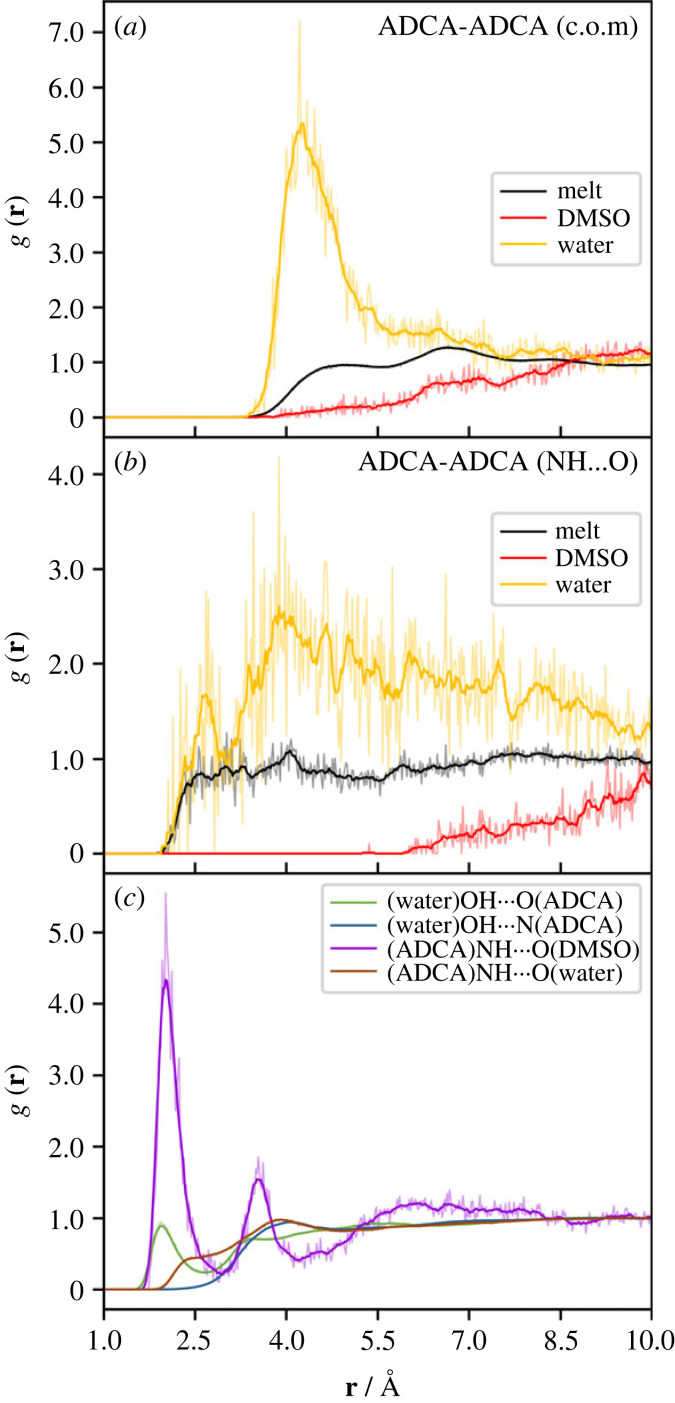
Radial distribution function (RDF) g(**r**) averaged over the entire 1 ns trajectory for the liquid ADCA and ADCA/solvent molecular dynamics simulations at 500 K. Semi-transparent lines represent the raw RDFs, while solid lines are the moving averaged RDFs calculated with a window-size of 10. (*a*) ADCA-ADCA g(**r**) as a function of the centre-of-mass (c.o.m.) distances **r** (in Å); (*b*) same as (*a*) but considering the distance NH^…^O among the ADCA molecules; (*c*) different atom-atom RDF of ADCA-solvent and solvent-ADCA distributions.

The purpose of classical Molecular Dynamics (MD) simulations was to gain insight into the solute-solvent molecular recognition process. Both liquid ADCA above its melting point (220–225°C) and ADCA solutions at room temperature were considered, with each simulation lasting 1.0 ns. Figure 4*a* shows the radial distribution functions (RDFs, g(**r**)) obtained by averaging over the whole trajectory of the melt phase at *T* = 500 K. A prominent self-solvation shell is observed at a centre of mass distance of ∼6.5 Å (figure 4*a*, black line). Another shoulder, with g(**r**) approximately 1, is also evident at approximately 4.5 Å. These peaks are consistent with the conformational constraint set by the E configuration of the double bond, which imposes an elongated shape of the backbone and hinders the formation of densely packed arrangements. Accordingly, hydrogen bonds are weak, with the amide carbonyl groups acting as the primary acceptor at H···O distance of approximately 2.5–3.0 Å. As expected, no significant H···N close contacts are observed (figure 4*b*, black line).

More intriguing results are found when considering MD simulations with explicit solvents. Figure 4 also illustrates the ADCA-ADCA and ADCA-solvent g(**r**) distributions in the two MD simulations involving water and DMSO as solvents. The first evidence is the complete absence of peaks in the centre-of-mass (c.o.m.) ADCA-ADCA g(**r**) for the DMSO case (red lines). This means that the molecules are surrounded by solvent molecules and kept apart from each other. On the contrary, the RDF distributions for water (figure 4*a*, yellow line) reveals the presence of first self-neighbors in the range of 4–5 Å. The NH_2_···O ADCA-ADCA hydrogen bond contacts (figure 4*b*) exhibit a similar trend, with no discernible peak in DMSO.

Figure 4*c* compares the hydrogen bond propensity of ADCA with water and DMSO. The curves are clearly asymmetric. Water acts as an effective hydrogen bond donor towards the amide oxygen atoms (but not nitrogen atoms) as evidenced by a weak peak around approximately 2 Å (green curve in figure 4*c*). However, water does not act as a hydrogen bond acceptor for the NH groups of ADCA (figure 4*c*, brown curve). The reason is that solvent-solvent OH···O hydrogen bonds are considerably stronger, due to the better donor/acceptor properties of water (electronic supplementary material, figure S4 SI). Conversely, ADCA sets up several short NH···O contacts with the S = O oxygen atom in dimethyl sulfoxide (purple curve in figure 4*c*), which does not bear other competing groups.

The liquid ADCA shows a large and negative total energy (kinetic + potential) of −334.3 kJ mol^–1^, which is comparable to that of the ADCA/DMSO solution (−336.93 kJ mol^–1^, see electronic supplementary material, table S5 for details). The total energy of the ADCA/water solution is one order of magnitude smaller, with values of −38.59 kJ mol^–1^. This neatly indicates that kinetic energy contributions are prevalent in this environment. On the contrary, the higher affinity of ADCA for DMSO mirrors energy contributions in larger potential, also in agreement with the experimental evidence. This is made even more evident by comparing the normalized cohesion energies per ADCA molecule, as estimated just from the potential energy contributions [11]. Table 1 summarizes the contributions obtained from the corresponding trajectory averages. Dispersive (van der Waals) interactions are considerably less significant in DMSO and negligible in water, as expected for these small molecules with a limited number of polarizable electrons. On the other hand, electrostatic (Coulomb) interactions are consistently dominant in determining the self-recognition of the solute. This is in line with the presence of a polar form in the liquid phase. Table 2 summarizes the distribution of ADCA conformers (figure 3) in the equilibrated liquid, obtained as time-average of the entire trajectories.

**Table 1.** Intermolecular contributions to the potential energy of the liquid ADCA and ADCA solutions with DMSO and water (cohesion energies). Values are given in kJ mol^−1^ per molecule. Estimated standard deviations are given in parentheses.

		liquid ADCA	ADCA/DMSO	ADCA/water
ADCA-ADCA	Coulomb	−405.1(1)	−380.6(1)	−383.1(1)
	Dispersion	−36.36(1)	−3.24(8)	−8.1(2)
	Total	−442.2(1)	−383.9(1)	−391.3(2)
ADCA-Solvent	Coulomb	—	−4.87(2)	−1.282(4)
	Dispersion	—	−3.47(1)	−0.341(2)
	Total	—	−8.33(2)	−1.624(4)
Solvent-Solvent	Coulomb	—	71.66(1)	−24.055(9)
	Dispersion	—	−27.697(8)	3.702(3)
	Total	—	43.96(1)	−20.35(1)

**Table 2.** Distribution of ADCA conformers (percent values) in different chemical environments, averaged over 1 ns-long trajectories. The estimated standard deviations are given in parentheses. Conformational labels are defined according to the mutual arrangement of the two O–C–N = N dihedral angles (figure 3), with a tolerance of ± 20°: T = 180 ± 20°, C = 0 ± 20°. ‘Other’ collects all the arrangements that do not fall within these limits.

system	TT	CC	M	other
liquid ADCA	0.31(1)	31.8(4)	23.4(4)	44.5(2)
ADCA/DMSO	0.0	10.78(1)	44.1(4)	45.1(4)
ADCA/water	0.0	10.2(1)	40.7(4)	49.1(4)

Polar conformations, that is, those exploiting one O–C–N = N dihedral at 180 ± 20° and the other one at 0 ± 20°, are always abundant. More precisely, on average, every ADCA molecule spends over 40% of its time adopting a polar conformation in solution, while this percentage is halved in the melt. Combined, the CC-like and M-like (figure 3) conformers account for more than 50% of the molecules in the liquid. It is expected that CC is present, as it is also the form that is found in the crystal. Another important piece of evidence from table 1 is that the strongest solute–solvent interactions occur in DMSO, again primarily driven by electrostatic forces. For the sake of comparison, the ADCA-solvent term is more than 80% smaller in water. At the same time, solvent-solvent potential contributions in DMSO are positive, indicating that the solvent prefers to interact with the solute rather than with itself. However, this is no longer true in water, where both solvent and solute molecules show a preference for interacting with their own kind. It is worth noting that solute-solvent interactions are consistently attractive, as they should be. The key distinction lies in the relative strength of interactions between different chemical species.

**Figure 5.**
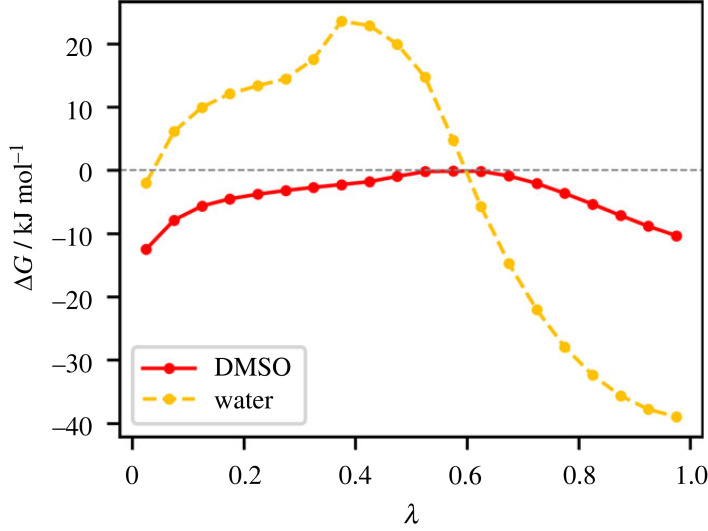
Gibbs free energy differences among neighbouring Hamiltonians in the BAR procedure for solubilization of one molecule of ADCA in two different solvents (red solid line: DMSO, yellow dashed line: water). Each *λ* value corresponds to a 1 ns-long simulation, where the Hamiltonian is changed by progressively (0.05, 5% per step) switching on solute-solvent interactions. The horizontal dashed grey line marks ΔG = 0 kJ mol^–1^. Lines serve only as guides for the eye.

Another factor that is worth investigating is the hydrophobic effect. The latter arises from the unfavourable entropy contribution associated with bulk water, which needs to restructure its hydrogen bond network to allocate a solute. To gain insight into the solvation thermodynamics of ADCA, we applied the Bennet Acceptance Ratio (BAR) technique [41] to the three solute–solvent molecular dynamics trajectories presented so far (see Methods for details). Results are shown in figure 5 and electronic supplementary material, table S6. The *λ* symbol represents an operational parameter that corresponds to the progressive ‘activation’ of the intermolecular electrostatic and dispersive potentials between solute and solvent. A value of *λ* = 0 corresponds to the least perturbed solvent (no interactions with the solute), while a value of *λ* = 1 indicates that the solute can fully interact with the solvent through both electrostatic and dispersive interactions. Intermediate *λ* values describe fictitious intermediate states, where solute-solvent electrostatic and dispersive interactions are only partially active. Each step of *λ* implies an increase of 5% of the solute–solvent interactions, until they are 100% active at *λ* = 1. In figure 5, each ΔG point represents the difference between contiguous *λ* points, i.e. between neighboring Hamiltonians.

The cumulative sum of Δ*G* values over the *λ* domain provides the total free energy change (Δ*G*_tot_) for the solvation process per solute molecule. In water (yellow points in figure 5), the addition of ADCA is initially endergonic (Δ*G* > 0), indicating that the process is thermodynamically unfavourable. During the early *λ* steps, ADCA poorly interacts with water, and the electrostatic and hydrogen bond contributions do not provide a sufficiently strong enthalpic term to compensate for the entropy loss of solvent. Consequently, the hydrophobic effect prevails. However, when the fraction of active electrostatic interactions increases above 60% (*λ* > 0.6), the interaction between ADCA and the water molecules becomes more favourable. The stabilizing contribution from these interactions eventually overwhelms the unfavourable entropic term. As a result, the overall Gibbs free energy balance becomes exergonic, and solvation becomes spontaneous (ΔG_tot_ < 0, see electronic supplementary material, table S6). By contrast, liquid DMSO lacks a structured hydrogen bond network that can be perturbed upon addition of the solvent. Consequently, the solvation process is exergonic even in the first steps of *λ*, when solute-solvent interactions are weak. For these reasons, the total ΔG of solvation per molecule (Δ*G*_tot_) is significantly more negative in DMSO compared to water (Δ*G*_tot_(DMSO) = −84.0(5) kJ mol^–1^; Δ*G*_tot_(water) = −57.01(5) kJ mol^–1^; see electronic supplementary material, table S6). A first conclusion is that the hydrophobic effect is indeed active in water, but it is not the most important factor governing the solubility behaviour of ADCA, as the overall ΔG for solubilization of a single ADCA molecule is always exergonic. The reason is that the interactions of individual solute molecules with water are attractive and compensate for unfavourable entropic effects. Again, this is likely due to the existence of polar forms of ADCA in solution and the formation of water–solute OH···O hydrogen bonds. However, despite these favourable solute-solvent interactions, water still exhibits a preference for self-interactions, resulting in poor solubility for ADCA in this solvent (see also electronic supplementary material, figure S4 SI). The most significant thermodynamic driving force for solubilization is still provided by DMSO.

### 3.4. Vapour-solution and solid-solution solvation thermodynamics

The analysis of the relative standard solvation entropy of ADCA, Δ_solv_S°, provides further insights into the solubility of ADCA in water and DMSO. The procedure developed by Sacchi *et al*. [14] was employed (§2.2), along with the same approximations (electronic supplementary material, section S3 SI). The results are summarized in table 3. In table 3, ‘vap, solv’ describes the vapour → solution process and ‘sol,solv’ the solid → solution one. The vapour-solution solvation free energies are large and negative for both water and DMSO, with a slight preference for DMSO. This is due to the generally favourable solute-solvent interactions. Moreover, these findings are consistent with the results observed from the BAR analysis of previous molecular dynamics simulations and indicate that the transfer of ADCA from gas phase to either water or DMSO is exergonic (Δ_vap,solv_G* < 0). On the contrary, the solvation (solid -> solution) free energies are both positive (Δ_sol,solv_G* > 0), with Δ_sol,solv_G*(water) > Δ_sol,solv_G*(DMSO). In other words, when the starting point is the crystal, the solubilization process is slightly endergonic for DMSO and almost three times more endergonic for water. This result is in line with the significant decrease in ADCA solubility observed in water.

**Table 3.** Approximate thermodynamic state functions at T∼20°C, as estimated by quantum simulations and experimental solubilities (see Methods and electronic supplementary material, section S3).

solvent	Δ_vap,solv_G*/kJ mol^–1^	Δ_sol,solv_G*/kJ mol^–1^	Δ_sol,solv_*S*^0^/J K^–1^ mol^–1^
water	−63.5	24.0	131.1
DMSO	−66.7	9.3	170.8

On the contrary, the solvation entropy is large and positive for both water and DMSO, with a larger contribution for DMSO. As the solubilization process is endothermic (§3.1), the thermodynamic driving force for ADCA dissolution is primarily entropic. The destruction of the crystal structure of ADCA contributes to the entropy gain. The lower entropy gain observed in water may be attributed to the lower interaction propensity of ADCA with water, which potentially preserves the water hydrogen bond network and explains why the hydrophobic effect plays a minor role (§3.3).

### 3.5. Molecular Dynamics: three-component nanodroplet systems

To explain the solubility results and to elucidate the solubility behaviour of ADCA in the presence of both DMSO and water, three-component Molecular Dynamics (MD) simulations employing a binary solvent mixture were carried out. Two nanodroplet models, namely Water-DMSO-ADCA (WDA) and DMSO-water-ADCA (DWA), were employed (see Methods and electronic supplementary material, section S4.1 SI).

WDA represents a nanodroplet of ADCA/DMSO solution embedded in bulk water, while DWA probes the reverse configuration (ADCA/water in bulk DMSO). The idea was to check whether a minimum quantity of DMSO could effectively solvate ADCA in the presence of water molecules, or if DMSO or water clusters could survive in the chemical environment provided by the other solvent.

The conformer distributions in the nanodroplets mirror those of the corresponding bulk liquids, with the polar M-like arrangement accounting for approximately 46% of the population in WDA and approximately 41% in DWA. Electronic supplementary material, table S7 shows the components of the average interaction energies (kJ mol^–1^) per ADCA molecule. In general, electrostatic contributions (E_el_) outweigh the Lennard-Jones ones (E_LJ_), reflecting the high polarity of DMSO or the proticity of water. Moreover, the average energies are consistently more negative (i.e. more stabilizing) for ADCA–DMSO interactions, regardless of the environment (DWA or WDA). The effect is particularly evident in DWA, where there is an excess of DMSO, but it is already appreciable in WDA, where water molecules exceed DMSO by a ratio of 11 to 1. ADCA-DMSO interaction energies are also always more negative than the DMSO-water ones, confirming that a preferential thermodynamic driving force toward the association of ADCA with DMSO exists also in the presence of a majority of water. It is important to note, however, that water significantly interacts with ADCA as well. A non-negligible estimate of E_el_ = –49 kJ mol^–1^ per molecule is appreciable for ADCA–water interactions, which is comparable in magnitude with the same quantity for the water self-interaction (electronic supplementary material, table S7).

**Figure 6.**
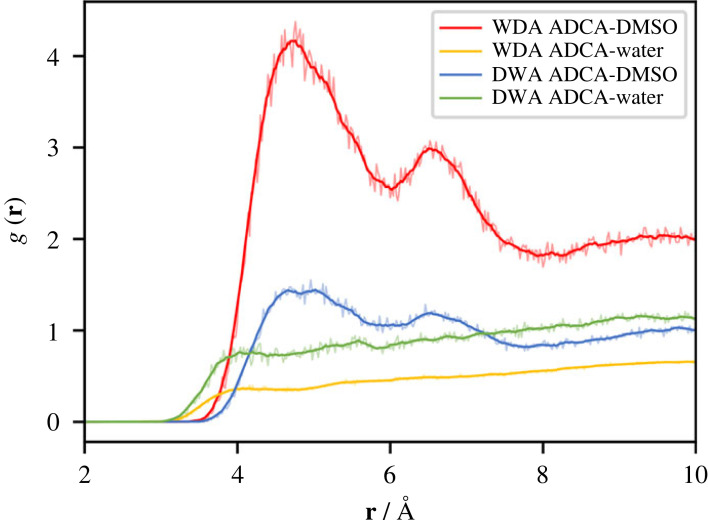
Radial distribution functions (RDFs) between the ADCA and solvent centre of mass (c.o.m) in either the WDA or the DWA environment (see text). The absolute values of the WDA and DWA curves are not directly comparable due to the different number of molecules and different dimensions of the simulation boxes. The semi-transparent lines are the raw radial distribution functions, while the solid curves depict their moving averages calculated with a window-size of 10.

Figure 6 shows the time-averaged RDFs, g(**r**), for the molecular centres of mass of the molecules in WDA and DWA. Interestingly, in both solvents, ADCA retains the same solvation structure primarily consisting of DMSO molecules (red and blue curves in figure 6). Two distinct peaks are clearly visible, corresponding to the first two shells of solvation, located at approximately 4.6 and 6.5 Å. On the contrary, the corresponding g(**r**) curves for water (yellow and green lines in figure 6) are very poorly structured and consistently remain below 1.0. This indicates that in both systems the probability of finding a water molecule close to ADCA is significantly lower compared to DMSO, even though it is not completely zero.

**Figure 7.**
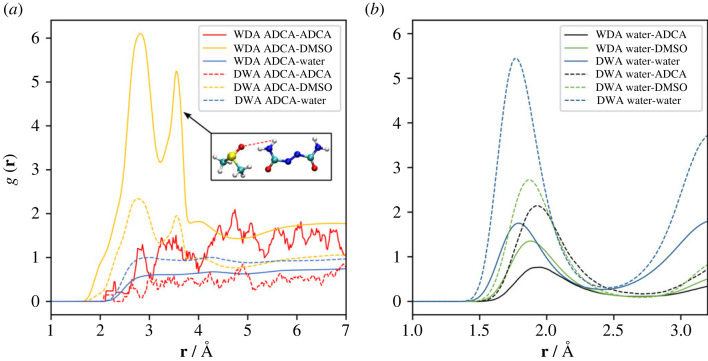
Running average of the radial distribution function g(**r**) of relevant H···O hydrogen bonded contacts. (*a*) WDA (solid lines) and DWA (dashed lines) environments (see text), NH···O interactions between the terminal NH_2_ groups of ADCA and oxygen acceptors belonging to itself (red), DMSO (yellow), and water (blue). (*b*) WDA (solid lines) and DWA (dashed lines) environments, OH···O interactions between water and oxygen acceptors belonging to ADCA (black), DMSO (green) and water itself (dark blue). The absolute values of the WDA and DWA curves are not directly comparable due to the different number of molecules and different dimensions of the simulation boxes. A window-size of 10 points was used to estimate the moving average.

The analysis of the g(**r**) functions for the H···O hydrogen bonded (HB) contacts (figure 7) further supports the previous conclusions. The nitrogen N atoms in ADCA are not considered in this analysis, owing to their poor ability to serve as HB acceptors. On the contrary, ADCA–NH_2_···O–DMSO interactions are frequently observed (yellow curves in figure 7*a*) and are structured into two peaks at ∼2.8 Å and ∼3.6 Å. These peaks represent the signature of the same HB interaction. The reason is that the sharper peak at higher distance is due to the proximity of the S = O oxygen atom of the solvent to the second hydrogen atom of the terminal NH_2_ moiety (figure 7*a*, inset).

On the other hand, no significant ADCA–NH_2_···O–ADCA interactions can be recognized (red curves in figures 7*a*). Instead, water predominantly forms hydrogen bonds with itself (blue curves in figure 7*b*) and, to a lesser extent, with DMSO (green curve in figure 7*b*). The interactions between water and DMSO involve direct bonds with average H···O distance below 2.0 Å. However, water can also establish some interaction with the ADCA, albeit weaker, as evidenced by the presence of longer HOH···O–ADCA contacts at approximately 2.0 Å (black curves in figure 7*b*). This behaviour can be explained considering that DMSO cannot saturate the HB acceptor propensity of amide groups in ADCA, since it lacks HB donors. Conversely, water is a much more effective HB donor and can favourably interact with all the available C = O and S = O groups. The result is that some water–ADCA interactions are unavoidable, even when a majority of DMSO is present.

Two important points can be highlighted. First, water can compete with ADCA to saturate the hydrogen bond valence of the S = O groups in DMSO. This means that water molecules can readily form hydrogen bonds with the DMSO, potentially reducing the availability of these molecules to interact with the solute. Second, the proximity of water and DMSO in the solvation environment may lead to a dielectric shielding effect on the electrostatic interactions, reducing the strength of electrostatic interactions between DMSO and ADCA. Overall, these two effects can collectively result in a reduction of the mutual affinity between DMSO and ADCA. This is consistent with the observed antisolvent behaviour of water with respect to ADCA/DMSO systems (§3.1).

### 3.6. Water-DMSO-ADCA models for high DMSO dilutions

**Figure 8.**
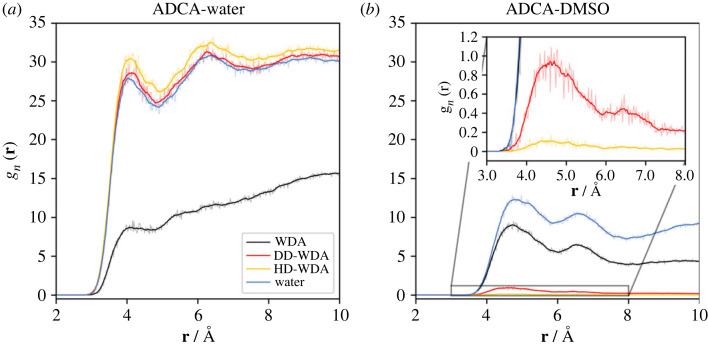
(*a*) Number density distributions, g_n_(**r**), of molecular centres of mass (c.o.m.) for ADCA-water pairs for various models at equilibrium. Black: WDA; red: DD-WDA; yellow: HD-WDA; blue: ADCA/solvent solution. (*b*) As (*a*), for the ADCA-DMSO interactions. The inset shows a zoom of the DD-WDA and HD-WDA curves. Semi-transparent solid lines are the raw number density distributions, while solid lines represent their moving averages calculated using a window-size of 10.

**Figure 9.**
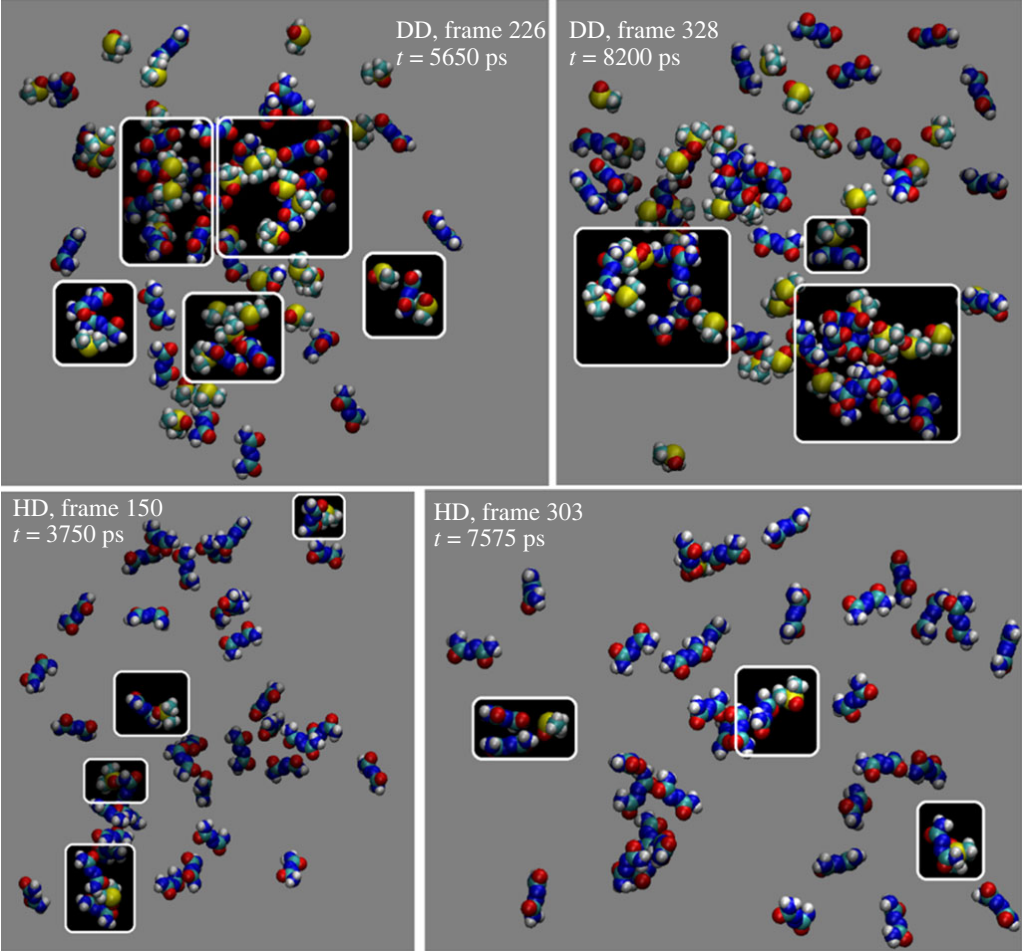
First row: representative examples of ADCA-DMSO transient aggregates (white boxes) in the DD-WDA liquid (2% DMSO). The pictures are snapshots taken from the whole 10 ns-long trajectory. Second row: same, for the HD-WDA liquid (0.2% DMSO). Individual atoms are shown as van der Waals spheres, with the usual colour codes (S: yellow; O: red; N: blue; H: white and C: cyan). The void space is filled by water molecules, which are omitted for clarity.

Two additional water-DMSO-ADCA (WDA) models, namely DD-WDA and HD-WDA (see Methods and electronic supplementary material, section S4.1 SI), infer the behaviour of the solute when a minority (either 2 ot 0.2%) of DMSO is present in an aqueous environment. The purpose is to investigate the strength of DMSO-ADCA interaction in a high dielectric medium, checking whether stable DMSO–ADCA adducts may persist when water is the main solvent. Figure 8*a* reports the corresponding number density distribution g_n_(**r**) for the centres of mass of the new models. As expected, ADCA is preferentially surrounded by water molecules in both mixtures. The reason is the significantly higher abundance of water compared to ADCA and DMSO, with water prevailing by a factor of over 200. Figure 8*b* focuses on the ADCA-DMSO interactions. The g_n_(**r**) functions are now normalized to express the number density of centre-of-mass contacts. This normalization allows for a better understanding of the frequency of ADCA-DMSO contacts as the DMSO concentration decreases. The red (DD) and yellow (HD) curves in figure 8*b* show the same features attributable to direct ADCA–DMSO solvation effects. The g_n_(**r**) blue curve in figure 8*b* shows peaks at ∼4.5 Å and 6.5 Å, indicating the presence of ADCA-DMSO contacts. However, no significant evidence of persistent solute-DMSO clusters is appreciable. Visual inspection of the trajectories, as shown in figure 9, reveals that ADCA-DMSO aggregates are indeed continuously formed and dissolved, while hydrogen bonds are exchanged with water.

In conclusion, the simulations indicate that even at very low DMSO content, close DMSO-ADCA contacts are formed. However, these contacts are not stable, and they are continuously broken due to the water competition for OH···O = S hydrogen bonds and thermal smearing. This is in line with the lower strength of NH···O = S bonds, as ADCA bears just weak NH hydrogen bond donors. It is likely the driving force for this kind of intermolecular association can be attributed to the strong electrostatic interactions between the polar form of the solute and DMSO, as both molecules have higher dipole moments compared to water.

Accordingly, the average interaction energies per solute molecule for ADCA–DMSO remain negative in both the DD-WDA (E_el_ = −5.7 kJ mol^–1^; E_LJ_ = −4.2 kJ mol^–1^) and HD-WDA scenarios (E_el_ = −0.6 kJ mol^–1^; E_LJ_ = −0.5 kJ mol^–1^), despite being one or two orders of magnitude lower than in the WDA model (electronic supplementary material, table S7). ADCA preferentially interacts with water in both the DD-WDA (E_el_ + E_LJ_ = −130.3 kJ mol^–1^) and HD-WDA (E_el_ + E_LJ_ = −136.0 kJ mol^–1^) environments, as it should be. The same is true for DMSO, which is very efficiently solvated by a water atmosphere.

In agreement with experimental outcomes, a water solution containing a small amount of DMSO should behave as pure water. Actually, the frequency of ADCA-DMSO contacts decreases as the dilution of DMSO increases, supporting the conclusion that the presence of DMSO has minimal impact on the overall thermodynamic behaviour of the solvent mixtures.

## 4. Conclusion

In this work, a systematic investigation of the solubility of azodicarbonamide (ADCA) in DMSO/water mixtures was carried out for the first time. ADCA was confirmed to be poorly soluble in water and moderately soluble in DMSO. The reasons were elucidated through a combination of complementary quantum and classical calculations and can be summarized as follows:

— Quantum mechanics calculations show the presence of a polar form of the title compound (the M conformer), unreported to date. This conformer is populated in liquid ADCA and solutions, where it can be stabilized by favourable electrostatic interactions with the environment. This contributes to explain the known solubility of ADCA in polar solvents.
— Thermodynamics data obtained from solid-state quantum mechanics simulations highlight that the driving force for ADCA dissolution in water is primarily entropic.
— Molecular dynamics shows that the solvent proticity is another crucial factor for ADCA solubility: a good hydrogen bond (HB) acceptance propensity, in conjunction with a poor HB donor ability, are mandatory requirements. The reason is that a protic solvent with good HB acceptors like water has a very high propensity of setting up HBs with itself, hampering the formation of any HB-mediated solvation shell with the solute. On the contrary, DMSO cannot saturate the HB valences of the S = O group, which remains free to set up weak and fluxional HBs with the amide hydrogens of ADCA. The net outcome is that ADCA preferentially interacts with DMSO, which is more polar than water and completely aprotic, even in the presence of a large excess of water.
— Hydrophobic effects in aqueous environments play a minor role: ADCA is not able to significantly perturb the hydrogen HB network of liquid water, a fact that is mirrored by a lower solvation entropy drive with respect to DMSO. Very low concentration of DMSO in the ADCA/water system can generate transient solute-DMSO aggregates, which primarily arise from favourable electrostatic interactions between ADCA and DMSO. However, interactions between water and DMSO, as well as between ADCA and water, are far more probable. It is unlikely that such intermittent ADCA–DMSO contacts have a significant impact on the overall thermodynamics of the system.

## Data Availability

The relevant numerical data discussed in this paper are included in the Supplementary Materials. We entirely employed publicly available software codes to perform simulations, namely Gromacs and MiCMoS (free of charge), CRYSTAL and Gaussian (commercial). The raw computational data, including Molecular Dynamics trajectories, can be obtained free of charge from the public repository Zenodo (https://doi.org/10.5281/zenodo.10527743) [42]. Supplementary material is available online [43].
